# High expression of NR1D1 is associated with good prognosis in triple-negative breast cancer patients treated with chemotherapy

**DOI:** 10.1186/s13058-019-1197-x

**Published:** 2019-11-28

**Authors:** Hyelin Na, Jinil Han, Na-Lee Ka, Min-Ho Lee, Yoon-La Choi, Young Kee Shin, Mi-Ock Lee

**Affiliations:** 1 College of Pharmacy, Bio-MAX, and Research Institute of Pharmaceutical Sciences, Seoul National University, Seoul, 08826 Republic of Korea; 2 Gencurix, Inc, Seoul, 08394 Republic of Korea; 3 Department of Pathology, Samsung Medical Center, Sungkyunkwan University School of Medicine, Seoul, Republic of Korea

**Keywords:** NR1D1, TNBC, Chemotherapy, OS, DFS

## Abstract

**Background:**

Nuclear receptor subfamily 1, group D, member 1 (NR1D1) is a ligand-regulated nuclear receptor and transcriptional factor. Although recent studies have implicated NR1D1 as a regulator of DNA repair and proliferation in breast cancers, its potential as a therapeutic target for breast cancer has not been assessed in terms of clinical outcomes. Thus, this study aims to analyze NR1D1 expression in breast cancer patients and to evaluate its potential prognostic value.

**Methods:**

NR1D1 expression was analyzed by immunohistochemistry using an anti-NR1D1 antibody in 694 breast cancer samples. Survival analyses were performed using the Kaplan–Meier method with the log-rank test to investigate the association of NR1D1 expression with clinical outcome.

**Results:**

One hundred thirty-nine of these samples exhibited high NR1D1 expression, mostly in the nucleus of breast cancer cells. NR1D1 expression correlated significantly with histological grade and estrogen receptor status. Overall survival (OS) and disease-free survival (DFS) did not correlate significantly with NR1D1 expression in breast cancer patients regardless of whether they had received chemotherapy. Subgroup analysis performed according to molecular subtype of breast cancer showed a significant influence of high NR1D1 expression on OS (*P* = 0.002) and DFS (*P* = 0.007) in patients with triple-negative breast cancer (TNBC) treated with chemotherapy.

**Conclusions:**

High NR1D1 expression level had a favorable impact on OS and DFS in patients with TNBC treated with chemotherapy. NR1D1 should be investigated further as a possible prognostic marker in TNBC patients receiving chemotherapeutic treatment and as a target in the development of chemotherapeutic approaches to treating TNBC.

## Background

Breast cancer is the most common cancer and the main cause of cancer death in women worldwide [1]. It is a diverse and complex disease in terms of its histology and clinical outcomes. In recent decades, significant advances have been made in the molecular classification of breast cancer and in treatment strategies including prognostic prediction. The estrogen receptor (ER), progesterone receptor (PR), and erb-b2 receptor tyrosine kinase 2 (ERBB2, also known as human epidermal growth factor receptor 2 (HER2)), are representative molecular biomarkers that distinguish breast cancer subtypes, i.e.*,* ER^+^/HER2^−^, ER^+^/HER2^+^, ER^−^/HER2^+^, and ER^−^/HER2^−^ [2]. Among these types of breast cancer, ER^−^/HER2^−^ breast cancer, also known as triple-negative breast cancer (TNBC), because of the lack of ERα, PR, and ERBB2 expression, is the most aggressive subtype with advanced histological grade and poor clinical outcome despite appropriate treatment [3]. Because of the lack of appropriate targets, there is no specific systemic treatment such as endocrine therapy or HER2-targeted therapy for TNBC. At present, the basis of TNBC treatment is chemotherapy and radiotherapy, which are associated with serious side effects. Thus, the identification of new targets may provide benefits in the treatment of women with TNBC by minimizing the side effects.

One of the characteristics that make TNBC a more aggressive and malignant subtype is a defective DNA damage response system. For example, the incidence of the germline breast cancer 1 (*BRCA1*) mutations is relatively high in TNBC, and most *BRCA1* mutation-associated breast cancers are TNBC [3]. Similarly, defects in genes related to DNA damage repair and genome maintenance, such as the Fanconi Anemia Complementation (FANC) group genes and 8-oxoguanine glycosylase 1, have been reported in TNBCs [4, 5]. Given this strong association between TNBC and the defects in genes involved in DNA damage repair, understanding the DNA damage response system may provide important prognostic clues about TNBC.

Nuclear receptor subfamily 1, group D, member 1 (NR1D1), also known as REV-ERBα, is a ligand-regulated nuclear receptor and transcriptional factor that binds directly to specific DNA response elements and represses target gene transcription [6]. NR1D1 regulates diverse biological processes such as the circadian clocks, cellular differentiation, metabolism, immune responses, and behavior [7]. Several studies have reported that NR1D1 is closely associated with the pathophysiology of breast cancer. *NR1D1* is located in the *ERBB2* amplicon region of chromosome 17q12–21 and is thought to be part of the ERBB2 signature, which is associated with poor clinical outcome in breast cancer [8, 9]. A synthetic NR1D1 agonist, SR9011, suppresses the proliferation of breast cancer cells regardless of the molecular subtype of breast cancer [10].

We recently reported a newly identified function of NR1D1, namely impairment of proper DNA repair. In breast cancer cells, NR1D1 is recruited to DNA damage sites and therein interacts with poly (ADP-ribose) polymerase 1 (PARP1) and subsequently inhibits the recruitment of the DNA damage response complex including SIRT6, pNBS1, and BRCA1 [11, 12]. Although NR1D1 may provide therapeutic options for improving the outcome of chemotherapy in breast cancer patients, its potential as a therapeutic target for breast cancer has not been clearly assessed in clinical outcomes. Therefore, in the present investigation, we performed a retrospective study to investigate NR1D1 expression in breast cancer patients and to evaluate its potential prognostic value.

## Methods

### Breast cancer tissue samples and patient information

Primary breast carcinoma samples were obtained from Samsung Medical Center in Seoul, Korea, between 1995 and 2002. A total of 694 breast cancer patients were retrospectively investigated. This breast cancer cohort was from the Samsung Medical Center Breast Cancer Biomarker Study (SMC-BCBS) [13]. The clinicopathological data included age, tumor characteristics such as tumor size, lymph node (LN) involvement, LN metastasis, American Joint Committee on Cancer (AJCC) stage, and pathological stage. Adjuvant chemotherapy treatment and survival data were obtained from medical records (Table 1). Cases were classified into breast cancer subtypes, ER^+^/HER2^−^, ER^+^/HER2^+^, ER^−^/HER2^+^, and TNBC. The protocol was approved by the ethical committees of the institutional review board (IRB) of the Samsung Medical Center (IRB File No. 2017-11-078).

**Table 1:** Characteristics of breast cancer patients and their NR1D1 expression level

Characteristics	All patients		Low NR1D1		High NR1D1		
	*n* = 694		*n* = 555		*n* = 139		
	No.	(%)	No.	(%)	No.	(%)	*P* value^a^
Age at diagnosis (years)							0.199
≤ 50	420	60.5	343	61.8	77	55.4	
> 50	274	39.5	212	38.2	62	44.6	
Tumor size							0.077
T1	269	38.8	221	39.8	48	34.5	
T2	378	54.5	292	56.2	86	61.9	
T3 or T4	47	6.8	42	7.6	5	3.6	
LN involvement							0.813
N0	354	51.0	288	51.9	66	47.5	
N1	180	25.9	142	25.6	38	27.3	
N2	85	12.2	67	12.1	18	12.9	
N3	75	10.8	58	10.5	17	12.2	
LN metastasis							0.404
Negative	354	51.0	288	51.9	66	47.5	
Positive	340	49.0	267	48.1	73	52.5	
AJCC stage							0.210
I	168	24.2	142	25.6	26	18.7	
II	353	50.9	275	49.5	78	56.1	
III	173	24.9	138	24.9	35	25.2	
Histologic grade							*0.011*
1	76	11.0	69	12.4	7	5.0	
2	246	35.4	201	36.2	45	32.4	
3	323	46.5	246	44.3	77	55.4	
Unknown	49	7.1	39	7.0	10	7.2	
Estrogen receptor							*0.021*
Negative	282	40.6	213	38.4	69	49.6	
Positive	411	59.2	341	61.4	70	50.4	
Unknown	1	0.1	1	0.2	0	0.0	
Progesterone receptor							0.393
Negative	379	54.6	298	53.7	81	58.3	
Positive	314	45.2	256	46.1	58	41.7	
Unknown	1	0.1	1	0.2	0	0.0	
HER2 amplification							0.113
Negative	504	72.6	411	74.1	93	66.9	
Positive	190	27.4	144	25.9	46	33.1	
Breast cancer subtype							0.067
ER+/HER2−	334	48.1	281	50.6	53	38.1	
ER+/HER2+	97	14.0	74	13.3	23	16.5	
ER−/HER2+	92	13.3	69	12.4	23	16.5	
TNBC (ER−/HER2−)	170	24.5	130	23.4	40	28.8	
Undefined	1	0.1	1	0.2	0	0.0	
Adjuvant chemotherapy							0.649
No	85	12.2	70	12.6	15	10.8	
Yes	607	87.5	483	87.0	124	89.2	
Unknown	2	0.3	2	0.4	0	0.0	

^a^Chi-square test, *P* values less than 0.05 are considered as significant changes and marked in italics

### Immunohistochemical analysis

The immunohistochemical analysis was performed as described previously [13]. Cores from breast cancer tissues were obtained, and tissue microarray paraffin blocks were generated. According to a routine protocol, the sections were deparaffinized with xylene, hydrated in serial dilutions of alcohol, and then were incubated in a 0.3% hydrogen peroxide solution for 15 min to neutralize endogenous peroxidase activity. Next, the sections were microwaved in citrate buffer for antigen retrieval. The tissue sections were then incubated for 1 h at room temperature with a primary antibody against NR1D1 (H00009572-M02, Novus Biologicals LCC, Littleton, CO, USA) diluted to a final concentration of 1:1000. Subsequently, the tissue sections were washed and reacted with an anti-mouse secondary antibody conjugated with a horseradish peroxidase-labeled polymer (K4001, Dako, Glostrup, Denmark) according to the manufacturers’ instructions. The tissue sections were rinsed, and stained with liquid diaminobenzidine tetrahydrochloride, a high-sensitivity substrate-chromogen system (K3468, DAKO, Glostrup, Denmark) for 5 min. Counterstaining was performed with Meyer’s hematoxylin. The immunohistochemical staining was scored by pathologists based on the intensity of staining and percentage of stained tumor cells. The intensity of staining was scored as 0, negative; 1, weak; 2, moderate; and 3, strong. The percentage of stained tumor cells was quantified and scored from 0 to 4: 0, negative or few; 1, < 25%; 2, 25–50%; 3, 50–75%; and 4, > 75%. The percentage and the staining intensity were then multiplied in order to generate the immunoreactive score for each of tumor specimens. Immunoreactive scores 0, 1, 2, 3, 4, 6, 8, and 9 were classified as low NR1D1 expression, and score 12 was classified as high NR1D1 expression. The numbers of specimens in each immunohistochemical staining score were as follows: 0, *n* = 3 (0.4%); 1, *n* = 23 (3.3%); 2, *n* = 69 (9.9%); 3, *n* = 62 (8.9%); 4, *n* = 86 (12.4%); 6, *n* = 129 (18.6%); 8, *n* = 115 (16.6%); 9, *n* = 68 (9.8%); and 12, *n* = 139 (20.0%).

### Statistical analysis

Correlations between NR1D1 expression and clinicopathological characteristics were analyzed using the chi-squared test. Overall survival (OS) was defined as the time from the date of the primary surgery until the date of death or the last follow-up. Disease-free survival (DFS) was defined as the interval from the date of the primary surgery to the date of recurrence, which indicated locoregional recurrence, distant metastasis, or death from any cause. The Kaplan–Meier method was used for survival analysis, and the log-rank test was used to estimate the associations between variables and survival. A univariable logistic regression model was used to estimate the association of NR1D1 expression with clinicopathological factors. The multivariable Cox regression model was used to identify significant prognostic factors among the clinicopathological factors and NR1D1 expression. *P* values < 0.05 were considered as significant. The statistical analyses were performed using R software for statistical computing and graphics (http://r-project.org).

## Results

### Characteristics of breast cancer patients according to NR1D1 expression

**Fig. 1:**
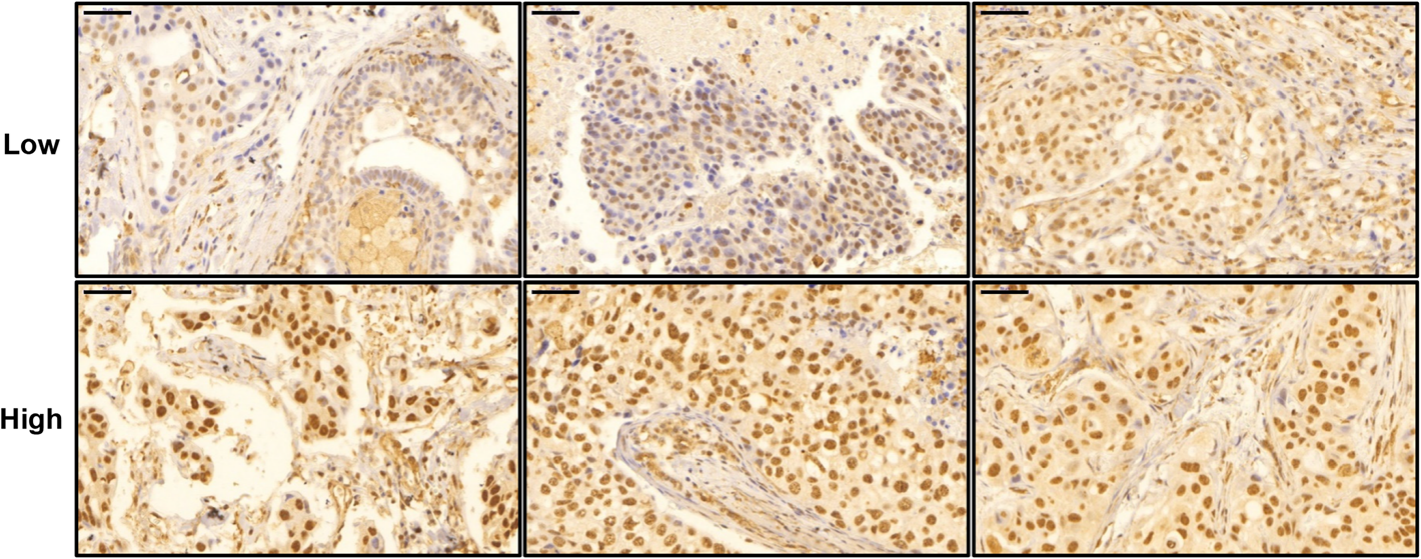
Representative tissue microarray specimens stained for NR1D1. Immunoreactivity of representative tumor specimens is shown. Low and high NR1D1 expression groups are defined in methods. The low NR1D1 expression ranges from immunoreactive scores 2 to 6 (left (P2xI1), middle (P3xI1), and right (P3xI2)), and the high NR1D1 expression shows immunoreactive score 12 (all, P4xI3). P, percentage of stained tumor cells; I, intensity of staining. Scale bar, 50 μm

We used immunohistochemistry to analyze NR1D1 expression in primary invasive breast cancer specimens. A total of 694 patients who had informative immunohistochemical results were included in this study. A representative tissue microarray stained for NR1D1 is shown in Fig. 1. NR1D1 signals were found predominantly in the nuclei of tumor cells in almost all samples. Immunohistochemical staining scores were evaluated and classified into low- and high-NR1D1 expression groups. In these patients, the high-NR1D1 expression group score was 12 (*n* = 139) and the low-NR1D1 expression group score was < 12 (*n* = 555). The clinicopathological characteristics of the patients in the study cohort are summarized in Table 1. Most of the patients (87.5%) were treated with adjuvant chemotherapy. We estimated the correlations of NR1D1 expression with clinicopathological factors. NR1D1 expression correlated significantly with the clinicopathological feature histological grade (*P* = 0.011) and ERα status (*P* = 0.021). However, the correlations with other parameters including adjuvant chemotherapy were not significant (Table 1).

### Survival analysis

**Fig. 2:**
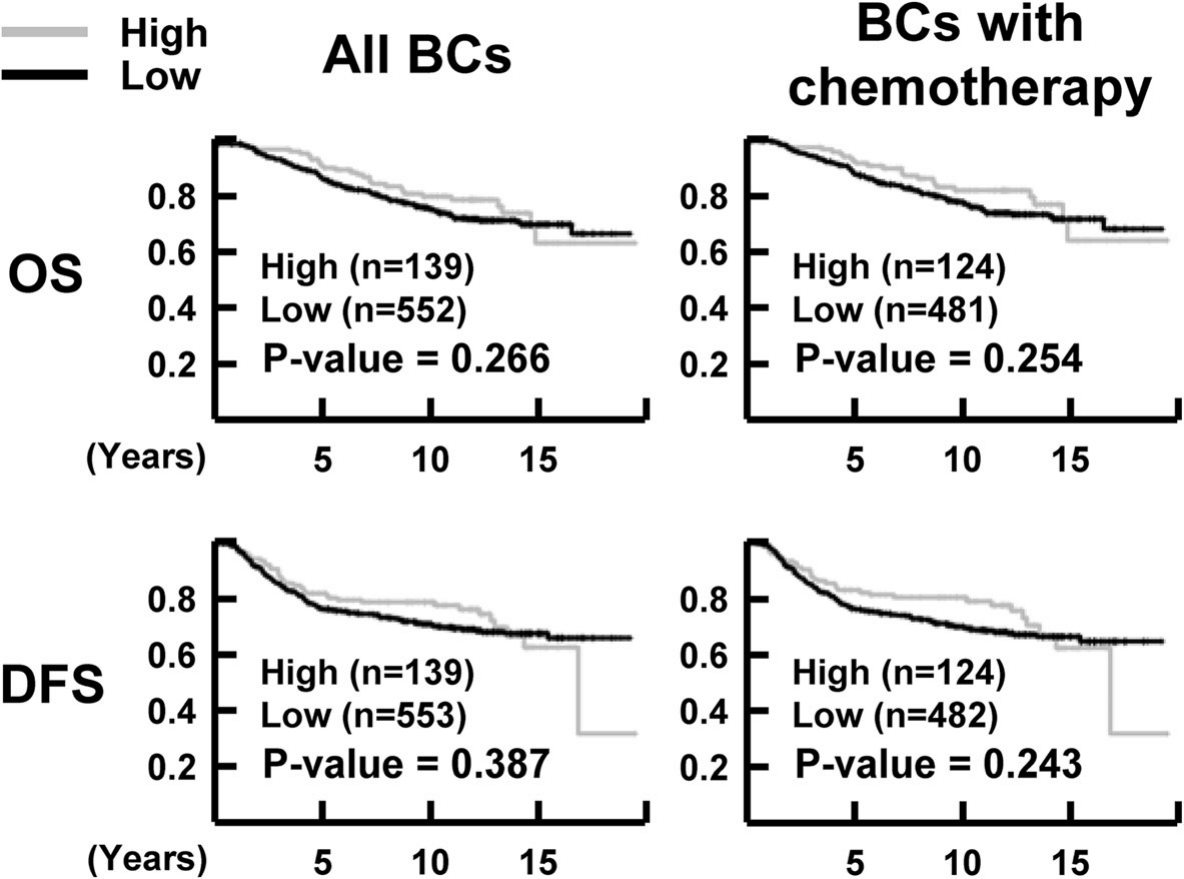
Association of NR1D1 expression with clinical outcome in breast cancer patients. Kaplan−Meier curves for OS and DFS according to NR1D1 expression in all breast cancer patients and in breast cancer patients receiving chemotherapy. Three or two patients without OS or DFS information, respectively, were excluded from the analysis

**Fig. 3:**
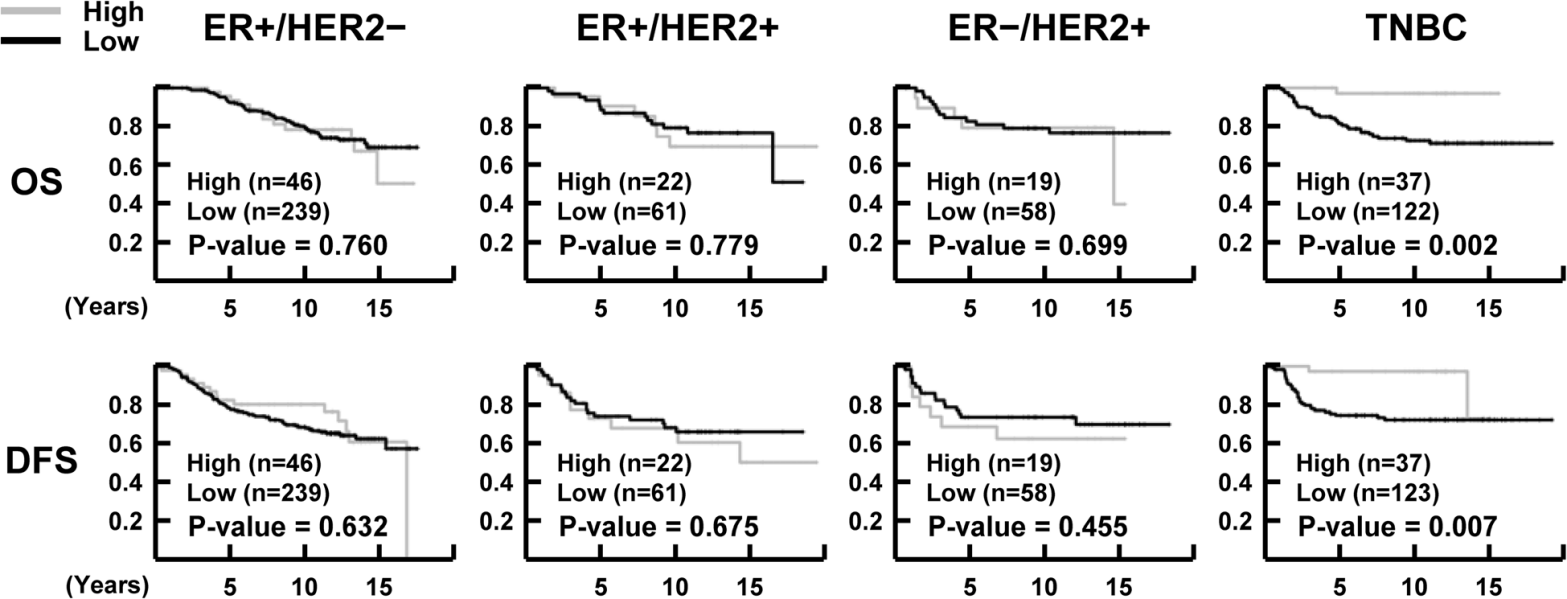
Prognostic significance of NR1D1 expression in TNBC patients treated with chemotherapy. Kaplan−Meier curves for OS and DFS according to NR1D1 expression in molecular subtypes of breast cancer patients treated with chemotherapy. A patient with no subtype information was excluded from the analysis

The median follow-up times for OS and DFS were 10.3 years (range, 0.1–19.5 years) and 9.4 years (range, 0.1–19.5 years), respectively. During the follow-up, 28.6% (198 of 692) of the patients had recurrence and/or metastasis, and 23.9% (165 of 691) of the patients died. To investigate the association of NR1D1 expression with clinical outcome, survival analyses were performed using the Kaplan–Meier method with the log-rank test. NR1D1 expression did not correlate significantly with OS (*P* = 0.266) or DFS (*P* = 0.387) in the breast cancer patients when all the samples were included in the analysis. In breast cancer patients who received chemotherapy, the OS (*P* = 0.254) and DFS (*P* = 0.243) did not differ significantly between groups with low or high NR1D1 expression (Fig. 2). Subgroup analyses according to molecular subtype of breast cancer in patients treated with chemotherapy showed significant influences of high NR1D1 expression on OS (*P* = 0.002) and DFS (*P* = 0.007) in TNBC patients who received chemotherapy (Fig. 3). These results suggest that high NR1D1 expression had a favorable effect on OS and DFS in TNBC patients treated with chemotherapy.

### Prognostic analysis

Finally, we evaluated the association of NR1D1 expression with survival of TNBC patients who received chemotherapy. In the univariate analysis, after chemotherapy, TNBC patients with high NR1D1 expression had a greater probability of an improved OS and DFS than did those with low NR1D1 expression. The factors that predicted poor OS and DFS based on the univariate analysis were larger tumor size, presence of LN involvement, presence of LN metastasis, and advanced AJCC stage in TNBC patients who received chemotherapy (Table 2). To investigate this in depth, multivariate analysis was performed using Cox proportional hazard models that included the factors that were significant or marginal significant in the univariate analysis. The LN involvement was excluded because of the redundancy to LN metastasis and the small size of each subgroup. Importantly, NR1D1 expression in TNBC patients who received chemotherapy was significantly related to improvements in OS and DFS in the multivariate analysis. Our results suggest that high NR1D1 expression is associated with chemotherapeutic benefits and may be an independent prognostic factor in patients with TNBC (Table 3).

**Table 2:** Univariate analysis of OS and DFS in the TNBC patients treated with chemotherapy

Variables	OS				DFS			
	No.	HR	95% CI	*P* value	No.	HR	95% CI	*P* value
NR1D1 expression								
Low	122	1.00			123	1.00		
High	37	0.09	0.01–0.63	*0.016*	37	0.17	0.04–0.73	*0.017*
Age at diagnosis								
≤ 50	109	1.00			110	1.00		
> 50	50	1.25	0.62–2.55	0.535	50	0.80	0.37–1.71	0.564
Tumor size								
T1	55	1.00			56	1.00		
T2	93	2.16	0.87–5.36	0.096	93	2.09	0.89–4.86	0.089
T3 or T4	11	7.63	2.46–23.70	*< 0.001*	11	4.58	1.34–15.68	*0.015*
LN involvement								
N0	93	1.00			94	1.00		
N1	36	1.03	0.36–2.94	0.950	36	0.77	0.28–2.10	0.607
N2	17	3.01	1.13–8.04	*0.028*	17	2.18	0.85–5.57	0.105
N3	13	15.26	6.46–36.05	*< 0.001*	13	7.99	3.23–19.79	*< 0.001*
LN Metastasis								
No	93	1.00			94	1.00		
Yes	66	2.79	1.37–5.66	*0.005*	66	1.77	0.90–3.46	0.099
AJCC stage								
I	42	1.00			43	1.00		
II	84	1.55	0.50–4.74	0.446	84	1.64	0.60–4.48	0.333
III	33	6.65	2.22–19.93	*< 0.001*	33	4.49	1.60–12.63	*0.004*
Histologic grade								
1	3	–			3	–		
2	31	1.00			31	1.00		
3	99	1.86	0.64–5.39	0.251	100	1.57	0.60–4.14	0.358
Unknown	26	–	–	–	26	–	–	–

*P* values less than 0.05 are considered as significant changes and marked in italics

*HR* hazard ratio, *CI* confidential interval

**Table 3:** Multivariate analysis of OS and DFS in the TNBC patients treated with chemotherapy

Variables	OS				DFS			
	No.	HR	95% CI	*P* value	No.	HR	95% CI	*P* value
NR1D1 expression								
Low	122	1.00			123	1.00		
High	37	0.09	0.01–0.65	*0.017*	37	0.16	0.04–0.68	*0.013*
Tumor size								
T1	55	1.00			56	1.00		
T2	93	0.76	0.14–4.13	0.753	93	0.73	0.14–3.79	0.705
T3 or T4	11	1.05	0.15–7.37	0.963	11	0.76	0.10–5.65	0.793
LN metastasis								
No	93	1.00			94	1.00		
Yes	66	0.97	0.27–3.57	0.967	66	0.64	0.19–2.19	0.474
AJCC stage								
I	42	1.00			43	1.00		
II	84	2.36	0.30–18.35	0.413	84	3.02	0.43–21.03	0.265
III	33	8.61	0.52–144.06	0.134	33	10.53	0.71–157.21	0.088

*P* values less than 0.05 are considered as significant changes and marked in italics

*HR* hazard ratio, *CI* confidential interval

## Discussion

Because of the lack of effective therapeutic targets, there is no specific systemic treatment for TNBC. Currently, TNBC treatment is typically based on chemotherapy and radiotherapy. Despite the standard chemotherapy regimens, TNBC patients respond differently to chemotherapy, ranging from early remission to worse OS [14, 15]. Thus, identification of new biomarkers that can be used to predict the response to conventional TNBC therapy may be beneficial in the treatment of TNBC. In this study, we found that high NR1D1 expression had a favorable impact on OS and DFS in patients with TNBC treated with chemotherapy.

The DNA damage repair system is often defective in TNBC patients, and monitoring of the DNA damage response is an important prognostic clue. In TNBC patients, the expression of DNA damage repair genes, such as xeroderma pigmentosum complementation group F, *FANC* group genes, *PARP1*, and *RAD51*, is associated with sensitivity to chemotherapy [16, 17]. We recently reported that NR1D1 is a crucial component of the DNA damage response, which may suggest that a high expression level of NR1D1 increases the susceptibility of TNBC to DNA damage induced by chemotherapeutic agents and ultimately leading to a better OS in TNBC patients [11]. Although our previous study showed the inhibitory function of NR1D1 in DNA repair in MCF7 cells (ER^+^/HER2^−^), this function was also demonstrated in other molecular subtypes including BT474 (ER^+^/HER2^+^) and MDA-MB-231 (TNBC) cells (data not shown). Interestingly, however, the prognostic effect of NR1D1 was exclusively found in TNBC and not in other subtypes. One possible explanation could be that gene variables that confer susceptibility to chemotherapy may vary according to molecular subtypes. For example, we showed previously that relative expression of proliferation-related and immune response-related genes, i.e., UBE2C, TOP2A, RRM2, FOXM1, MK167, and BTN3A2, provided a prediction value of adjuvant chemotherapy benefit for patients with ER^+^/HER2^−^ early breast cancer [18]. In ER^−^/HER^+^ subtype, immune-related genes, i.e., BTN3A2, CD2, and TRBC1, and invasiveness-related MMP11 were significantly associated with a prognosis of this disease [19]. Thus, TNBCs could be the most susceptible subtype that are being benefited from the high intratumoral expression of NR1D1 after treatment with DNA damaging chemotherapy.

Interestingly, several studies have reported that genetic polymorphism of *NR1D1* is associated with human diseases such as bipolar disorder and obesity [20–23]. The variations were located mainly in the first intron and in the 5′ untranslated region of *NR1D1*, which may cause differential expression of the gene, as shown in breast cancer patients. Thus, the role of genetic variations in *NR1D1* in the pathogenesis and progression of breast cancer as well as the chemotherapeutic responses may be an important issue. More detailed analyses of the polymorphisms and expression level of *NR1D1* together with genomic analysis for defects in DNA damage repair genes may provide prediction value for clinical outcomes of adjuvant chemotherapy in TNBC patients.

Because 10–20% of breast cancers are TNBC, identification of new targets that could maximize the efficacy but minimize the side effects of chemotherapy is an unmet need [24]. Recently, small molecules that can modulate the activity of NR1D1 and their potential as anticancer therapeutics were demonstrated [25]. SR9011, a synthetic ligand of NR1D1, has been reported to inhibit the proliferation of various breast cancer cell lines and to induce cell cycle arrest by suppressing cyclin A [10]. Because NR1D1 increases DNA damage induced by chemotherapeutic agents, the potential of combining chemotherapy with ligands of NR1D1 may be a therapeutic option for a more effective approach. GSK4112, a synthetic ligand of NR1D1, increased the chemosensitivity of breast cancer cells to doxorubicin [11]. In conclusion, our study suggests that the expression level of NR1D1 is both a prognostic marker for patients under chemotherapeutic treatment and a target for the development of chemotherapeutic approaches for the treatment of TNBC.

## Conclusions

Although NR1D1 implicates as a regulator of DNA repair and proliferation in breast cancers, its potential as a therapeutic target for breast cancer has not been assessed in clinical outcomes. Our data showed that high NR1D1 expression had a favorable impact on OS and DFS in patients with TNBC treated with chemotherapy. NR1D1 should be investigated further as a possible prognostic marker in TNBC patients receiving chemotherapeutic treatment and as a target in the development of chemotherapeutic approaches to treating TNBC.

## Data Availability

The datasets used and/or analyzed during the current study are available from the corresponding author on reasonable request.

## Abbreviations

AJCC: American Joint Committee on Cancer
BRCA1: Breast cancer 1
DFS: Disease-free survival
ER: Estrogen receptor
ERBB2: erb-b2 receptor tyrosine kinase 2
FANC: Fanconi Anemia Complementation
HER2: Human epidermal growth factor receptor 2
LN: Lymph node
NR1D1: Nuclear receptor subfamily 1, group D, member 1
OS: Overall survival
PARP1: Poly(ADP-ribose) polymerase 1
PR: Progesterone receptor
TNBC: Triple-negative breast cancer
